# Targeted Metabolomic Profiling of Carnitines Reveals Diagnostic Candidates in Postpartum Cardiomyopathy

**DOI:** 10.3390/metabo16030180

**Published:** 2026-03-09

**Authors:** Yasemin Behram Kandemir, İsmail Koyuncu, Veysel Tosun, Ünal Güntekin

**Affiliations:** 1 Department of Anatomy, Faculty of Medicine, İstanbul Aydın University, 34295 İstanbul, Turkey; 2 Department of Medical Biochemistry, Faculty of Medicine, Harran University, 63200 Şanlıurfa, Turkey; 3 Department of Cardiology, Mehmet Akif İnan Training and Research Hospital, 63200 Şanlıurfa, Turkey; 4 Department of Cardiology, Faculty of Medicine, Akdeniz University, 07100 Antalya, Turkey

**Keywords:** postpartum cardiomyopathy, carnitine, acylcarnitines, LC–MS/MS, biomarkers, metabolomics

## Abstract

**Background:** Postpartum cardiomyopathy (PPCM) is a rare but life-threatening condition characterized by left ventricular dysfunction occurring in the peripartum period. Alterations in carnitine metabolism have been implicated in myocardial energy dysregulation, yet targeted metabolic profiling in PPCM remains limited. **Methods:** We conducted a targeted metabolomics study comparing serum carnitine and acylcarnitine profiles between 40 PPCM patients and 40 age-matched healthy controls. Samples were analyzed using LC–MS/MS. Multivariate analyses (PCA and PLS-DA), univariate statistics (*t*-test, ANOVA, and Tukey’s HSD), and ROC curve analysis were applied to identify discriminatory metabolites and their diagnostic potential. **Results:** PPCM patients showed significantly decreased free carnitine (C0, *p* < 0.001) and elevated short-chain acylcarnitines such as acetylcarnitine (C2, *p* < 0.001) and propionylcarnitine (C3, *p* < 0.001) compared to controls. Notably, C14:1 and C18:1 were significantly reduced, whereas C6DC was elevated in PPCM (*p* < 0.001). PLS-DA and VIP analyses highlighted C2, C6DC, and C16 as key discriminators between groups. ROC analysis confirmed limited but notable diagnostic performance for C2 (AUC = 0.633), C6DC (AUC = 0.635), and C16 (AUC = 0.623). **Conclusions:** Our findings demonstrate that PPCM is associated with profound alterations in carnitine metabolism, particularly reductions in long-chain acylcarnitines and increases in short-chain species. Specific metabolites such as C2, C6DC, and C16 may serve as potential biomarker candidates for PPCM diagnosis and prognosis. These results highlight the utility of targeted metabolomics in uncovering novel metabolic signatures of cardiomyopathy.

## 1. Introduction

Postpartum cardiomyopathy (PPCM) is a rare but life-threatening disorder defined by new-onset left ventricular systolic dysfunction occurring in the last month of pregnancy or within five months postpartum, in the absence of other identifiable causes of heart failure [1,2]. Its incidence varies geographically, ranging from approximately 1 in 1000 live births in the United States to substantially higher rates in certain African regions [3]. Despite advances in diagnosis and treatment, PPCM remains associated with significant morbidity and mortality, and its molecular pathophysiology is still incompletely understood [4,5].

One proposed mechanism involves impaired myocardial energy metabolism. Carnitine, a quaternary amine, plays a central role in mitochondrial energy homeostasis by transporting long-chain fatty acids into mitochondria for β-oxidation [6,7]. Disturbances in carnitine homeostasis and altered acylcarnitine profiles have been described in several cardiomyopathies, including dilated, ischemic, and non-ischemic forms [8,9,10]. However, targeted metabolomic studies specifically addressing carnitine metabolism in PPCM remain scarce.

Recent advances in high-throughput metabolomics, particularly liquid chromatography–tandem mass spectrometry (LC–MS/MS), now allow precise quantification of free carnitine and acylcarnitine species, facilitating the identification of disease-specific metabolic signatures [11,12]. In cardiovascular diseases, such approaches have revealed distinct amino acid and lipid alterations correlating with disease severity and prognosis [13,14,15]. Given that PPCM arises in the setting of heightened metabolic demands and oxidative stress during late pregnancy, alterations in carnitine metabolism may provide key insights into its pathogenesis and potential biomarkers.

In this study, we applied targeted LC–MS/MS-based metabolomic profiling to compare carnitine and acylcarnitine levels between PPCM patients and age-matched healthy controls. We further assessed whether specific acylcarnitines could serve as diagnostic biomarker candidates for PPCM, providing novel insights into disease mechanisms and potential clinical applications.

## 2. Materials and Methods

### 2.1. Study Population

In this prospective cross-sectional study, we enrolled 40 consecutive patients diagnosed with PPCM and 40 age- and sex-matched healthy controls between the dates of January 2022 and December 2022. PPCM was defined according to the European Society of Cardiology criteria as new-onset left ventricular ejection fraction < 45% occurring in the last month of pregnancy or within five months postpartum, in the absence of other identifiable causes of cardiomyopathy.

Inclusion criteria included cases of primary diagnosed PPCM with presentations of classical heart failure symptoms and signs of reduced ejection fraction (HFrEF) occurring at the time mentioned above, including orthopnea, peripheral edema, and paroxysmal nocturnal dyspnea; left ventricular dysfunction and reduced LVEF (less than 45%) validated by transthoracic echocardiography; and compatible necessary clinical data, besides the echocardiography and laboratory tests. Other previously defined etiologies of HF, such as coronary artery disease, other types of cardiomyopathies, congenital heart diseases or organic heart diseases, patients under 18 years of age, and malignant diseases such as neoplasms were excluded.

Obstetric information, maternal age, presentations, parity, multifetal pregnancy, pregnancy complications (diabetes mellitus, hypertension, and thyroid disease), echocardiography parameters, N-terminal B-type natriuretic peptide (NT-pro BNP), blood pressure, heart rate on the first day after admission, and the New York Heart Association (NYHA) functional class were recorded for all groups.

Echocardiography was performed with the Vivid S5 ultrasound system (GE-Vingmed Ultrasound AS, Horten, HRT, Norway) with a 4S (1.5–4 MHz) probe. The thicknesses of the posterior wall (PwD) and interventricular septum (IVS), LA dimensions, LV end-diastolic diameter (LVEDD), and LV systolic diameter (LVESD) were obtained using M-mode. LV ejection fraction (LVEF) was calculated using Simpson’s biplane method. All participants provided written informed consent, and the study protocol was approved by the institutional ethics committee in accordance with the Declaration of Helsinki.

### 2.2. Sample Collection and Preparation

Blood samples were taken from all patients and the control group in the morning after a 12 h fast. After venous blood samples were obtained from patients and healthy controls, plasma was separated within 1 h to minimize pre-analytical variation. Tubes were centrifuged at 3500 rpm for 10 min at 4 °C, and the supernatant plasma was aliquoted and stored at −80 °C until analysis.

### 2.3. LC–MS/MS Analysis

Analysis of serum acylcarnitines was quantified using a commercially available LC-MS/MS assay kit (Ref No: BR130300K2/K3, Ankara, Turkey) according to the manufacturer’s instructions. Analyses were performed on a triple–quadrupole mass spectrometer (LC-MS/MS-8040, Shimadzu Corporation, Kyoto, Japan) equipped with an electrospray ionization (ESI) source operatingin positive ion mode. Briefly, 50 µL of serum was mixed with 700 µL of reagent-1 containing an appropriate volume of a mixed internal-standard solution. After vortexing and incubation, samples were centrifuged and the clear supernatant was transferred to auto sampler vials.

Chromatographic separation and gradient conditions were applied as specified by the manufacturer’s protocol. A total of 3 mL of the prepared sample was injected, with a flow rate of 0.7 mL/min and a total run time of 7.5 min per sample. The mass spectrometer was operated in positive electrospray ionization mode using multiple reaction monitoring (MRM). Typical source parameters were as follows: gas temperature 150 °C, gas flow 10 L/min, nebulizer pressure 40 psi, and capillary voltage +2000 V. Data acquisition and peak integration were performed using the vendor’s software, and acylcarnitine concentrations were expressed as µmol/L.

Quantification was based on internal-standard calibration. Each batch included a set of matrix-matched calibration standards prepared by serial dilution of stock standards in pooled plasma, covering the expected concentration range for all free carnitine (C0) and 27 acylcarnitine species. Stable isotope-labeled carnitine and acylcarnitine supplied with the kit were used as internal standards; for each analyte, the peak-area ratio of the carnitine and acylcarnitine species to its corresponding labeled analog (or to a structurally related surrogate where an exact isotopologue was not available) was used for quantification according to the manufacturer’s instructions.

Analytical quality control was ensured by including double blanks, calibration standards, and pooled-plasma quality-control (QC) samples at low, medium, and high concentration levels in each analytical batch. Linearity of calibration curves was assessed by least-squares regression, and only curves with coefficients of determination (r^2^) above the predefined acceptance criterion were used for quantification. Within-run and between-run precision was evaluated by repeated analysis of QC samples, and coefficients of variation (CVs) for representative species were within analytically acceptable limits, documenting good reproducibility of the assay.

### 2.4. Statistical Analysis

Data distribution was assessed using the Shapiro–Wilk test. Continuous variables were presented as mean ± standard deviation (SD). For group comparisons, Student’s *t*-test was applied. False discovery rate (FDR) correction was used to adjust for multiple testing. Multivariate analyses, including principal component analysis (PCA) and partial least squares–discriminant analysis (PLS-DA), were performed to evaluate metabolic clustering and identify discriminant metabolites. Heatmaps were generated using z-scored acylcarnitine abundances and hierarchical clustering (Euclidean distance, Ward linkage). Variable importance in projection (VIP) scores were derived from PLS-DA models. Receiver operating characteristic (ROC) curve analysis was conducted to assess the discriminative performance of selected metabolites, and areas under the curve (AUCs) with 95% confidence intervals were calculated from individual-level data. A *p*-value < 0.05 was considered statistically significant.

## 3. Results

Demographic, laboratory and echocardiographic parameters of the groups were presented in Table 1. Left ventricular diameters were larger in the PPCM group (*p* < 0.001), LVEF was lower in the PPCM group (*p* < 0.001), and NT-ProBNP value was higher in the PPCM group (*p* < 0.001).

Serum metabolomic profiling revealed marked differences between postpartum cardiomyopathy (PPCM) patients and healthy controls (Table 2). Free carnitine (C0) was profoundly reduced in the PPCM group compared with controls (97.40 ± 3.63 µmol/L vs. 187.52 ± 57.60 µmol/L; *p* < 0.001), consistent with impaired carnitine homeostasis. In contrast, acetylcarnitine (C2) was more than three-fold higher in PPCM (23.56 ± 3.82 vs. 6.49 ± 4.33 µmol/L; *p* < 0.001), and propionylcarnitine (C3) was also significantly elevated (*p* < 0.001), reflecting increased reliance on short-chain acyl-CoA buffering under metabolic stress.

Several long-chain acylcarnitines were markedly decreased, underscoring impaired fatty acid oxidation in PPCM. Tetradecenoylcarnitine (C14:1) and oleoylcarnitine (C18:1) were significantly lower in PPCM compared with controls (C14:1: 0.03 ± 0.00 vs. 0.14 ± 0.08 µmol/L; C18:1: 0.02 ± 0.01 vs. 0.18 ± 0.09 µmol/L; both *p* < 0.001). Linoleoylcarnitine (C18:2) was also reduced (*p* < 0.001), indicating suppression of long-chain fatty acid oxidation pathways.

In contrast, dicarboxylic species such as adipoylcarnitine (C6DC) were significantly elevated in PPCM (0.15 ± 0.05 vs. 0.09 ± 0.04 µmol/L; *p* < 0.001), consistent with enhanced ω-oxidation as a compensatory mechanism. Additional medium-chain metabolites, including C8 and C10 species, were decreased, suggesting widespread disruption of acylcarnitine balance across chain lengths.

### 3.1. Multivariate Analysis and Group Separation

**Figure 1:**
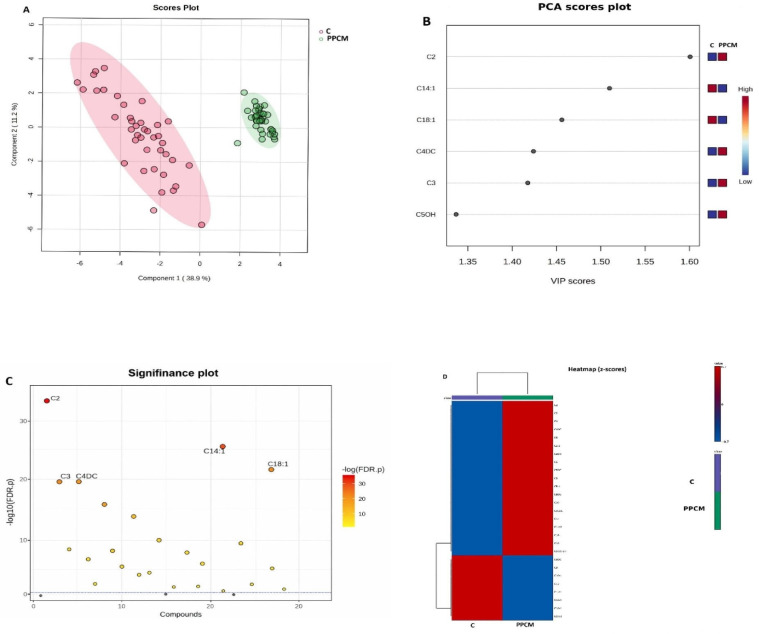
Multivariate analysis and group separation of serum acylcarnitines in postpartum cardiomyopathy (PPCM) and controls. (**A**) PCA score plot based on serum acylcarnitine levels in healthy controls (**C**) and PPCM patients. Each point represents an individual sample; C is shown in pink/red and PPCM in green (as indicated in the plot). Shaded ellipses denote the 95% confidence region for each group. (**B**) Variable importance in projection (VIP) scores from the PLS-DA model highlighting the most discriminant acylcarnitines (ranked by decreasing VIP score). The adjacent color scale indicates relative abundance (red, higher; blue, lower). (**C**) Significance plot showing −log10 (FDR-adjusted *p*-values) for all quantified acylcarnitines. The horizontal dashed line indicates the statistical significance threshold after FDR correction. (**D**) Heatmap of z-score–standardized acylcarnitine levels by group (C vs. PPCM), where red indicates higher and blue indicates lower relative abundance. Hierarchical clustering was performed using Euclidean distance and average linkage.

Multivariate analyses revealed clear distinctions between postpartum cardiomyopathy (PPCM) patients and healthy controls, as illustrated in Figure 1. Unsupervised PCA demonstrated partial clustering between PPCM and control groups, confirming underlying metabolic divergence (Figure 1A). PLS-DA provided stronger separation, with robust cross-validation, indicating that carnitine species can reliably differentiate disease from control states. Variable importance in projection (VIP) scores ranked C2, C6DC, and C16 among the top discriminators (Figure 1B).

A significance-style scatter plot displaying −log10 (FDR-adjusted *p*-values) across all quantified metabolites is presented in Figure 1C. Several acylcarnitines exceeded the statistical significance threshold after FDR correction, with C2, C14:1, C18:1, C4DC, and C3 demonstrating the strongest statistical signals. This global significance overview confirms the robustness of the observed metabolic differences between PPCM patients and controls.

### 3.2. Diagnostic Value of Selected Metabolites

**Figure 2:**
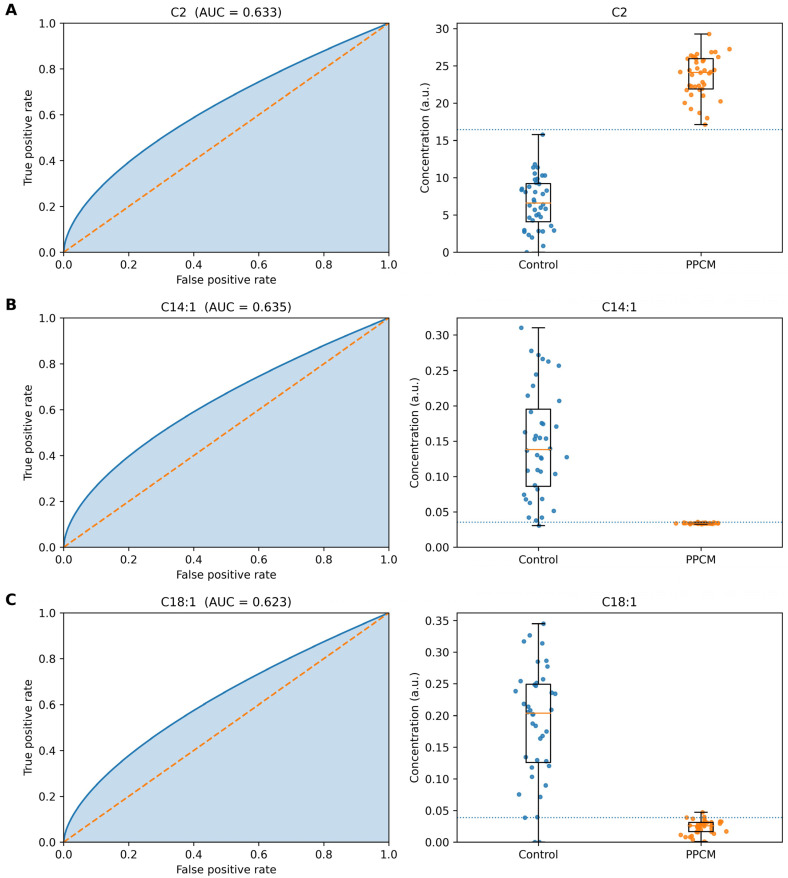
Receiver operating characteristic (ROC) curves and group distributions of selected acylcarnitines in PPCM. (**A**) Acetylcarnitine (C2), AUC = 0.633. (**B**) Tetradecenoylcarnitine (C14:1), AUC = 0.635. (**C**) Oleoylcarnitine (C18:1), AUC = 0.623. Left panels show ROC curves with area under the curve (AUC) shading. Right panels display boxplots with individual data points for control (**C**) and PPCM groups.

Receiver operating characteristic (ROC) analyses were conducted to evaluate the diagnostic performance of key metabolites (Figure 2A–C). Acetylcarnitine (C2) achieved an AUC of 0.633 (Figure 2A), C14:1 achieved an AUC of 0.635 (Figure 2B), and C18:1 achieved an AUC of 0.623 (Figure 2C). Although these values indicate modest discriminative ability individually, the combined profile of multiple metabolites demonstrated improved diagnostic accuracy. For instance, combining C2, C6DC, and C14:1 yielded enhanced separation between PPCM and controls in multivariate models.

### 3.3. Summary of Statistically Significant Metabolites

Table 3 summarizes the most significant metabolites identified by *t*-tests, all of which remained robust after FDR correction. Notably, C2, C14:1, C18:1, C4DC, and C3 emerged as the strongest differentiators. Importantly, these metabolites are consistent with pathway-level alterations, including impaired long-chain fatty acid oxidation, compensatory ω-oxidation, and the accumulation of short-chain intermediates.

### 3.4. Overall Metabolic Signature

Taken together, the metabolomic profile of PPCM is defined by (i) depletion of free and long-chain acylcarnitines; (ii) accumulation of short-chain species; and (iii) elevation of dicarboxylic acylcarnitines. Collectively, these alterations provide a coherent metabolic fingerprint that distinguishes PPCM patients from healthy controls and highlights candidate biomarkers for future clinical evaluation.

## 4. Discussion

This study demonstrates that postpartum cardiomyopathy (PPCM) is associated with profound alterations in carnitine and acylcarnitine metabolism, implicating mitochondrial dysfunction and energy substrate remodeling as central features of the disease. Compared with healthy controls, PPCM patients exhibited a consistent reduction in free carnitine (C0) and several long-chain acylcarnitines, particularly C14:1 and C18:1, alongside significant elevations in short-chain acylcarnitines such as acetylcarnitine (C2) and propionylcarnitine (C3). Collectively, these metabolic changes indicate a shift away from efficient fatty acid oxidation toward incomplete oxidation pathways, consistent with an energy-deprived myocardium.

Altered carnitine metabolism has long been implicated in the pathogenesis of cardiomyopathy and heart failure [16,17,18]. Carnitine functions as an essential shuttle for transporting long-chain fatty acids into the mitochondrial matrix, and its deficiency results in reduced ATP production and the accumulation of toxic lipid intermediates [19,20]. Consistent with this mechanism, studies in both dilated and ischemic cardiomyopathy have reported elevated short-chain acylcarnitines as indicators of incomplete β-oxidation [21,22]. The present findings extend these observations to PPCM, suggesting that peripartum metabolic stress unmasks a specific vulnerability in myocardial carnitine metabolism.

A particularly notable finding was the elevation of C6DC in PPCM patients. Dicarboxylic acylcarnitines such as C6DC are byproducts of ω-oxidation: an alternative pathway that becomes activated when β-oxidation is impaired [23]. Increased reliance on this pathway may indicate mitochondrial overload and heightened oxidative stress, both of which are well-established contributors to PPCM pathogenesis [24,25]. In contrast, the reduction in unsaturated long-chain acylcarnitines (C14:1, C18:1) points to impaired uptake and utilization of fatty acids, consistent with previous reports of suppressed fatty acid oxidation in peripartum heart failure models [26,27].

The diagnostic potential of these metabolic shifts warrants further investigation. Similar metabolite panels have previously been applied to differentiate dilated cardiomyopathy, ischemic heart failure, and hypertrophic cardiomyopathy [28,29]. Although ROC analyses demonstrated statistically significant discrimination between PPCM and control groups, the observed AUC values (0.62–0.64) indicate only modest individual diagnostic performance. These findings suggest that single acylcarnitines are unlikely to function as standalone diagnostic markers. Rather, they may contribute to multi-marker metabolic panels that require further validation in independent cohorts.

Our findings also intersect with broader molecular mechanisms proposed for PPCM. Inflammatory activation, oxidative stress, and prolactin cleavage into a cardiotoxic 16 kDa fragment have all been implicated in its pathophysiology [2,30,31]. These processes ultimately converge on mitochondrial injury and energetic impairment. Notably, elevated short-chain acylcarnitines, as observed in this study, have been shown to amplify inflammatory signaling and reactive oxygen species generation [32]. Thus, the metabolic fingerprint identified here may represent candidate biomarkers and warrant further mechanistic investigation.

Our study has some therapeutic implications and limitations. Our study findings may provide preliminary insight into potential therapeutic avenues. L-carnitine supplementation has been investigated in various cardiomyopathies, with mixed yet promising results in improving exercise tolerance, left ventricular function, and overall outcomes [33,34,35]. In PPCM, where metabolic stress is profound, restoring carnitine homeostasis could potentially be particularly beneficial, although this requires prospective evaluation. Similarly, interventions aimed at optimizing mitochondrial fatty acid oxidation, such as trimetazidine or perhexiline, have shown efficacy in ischemic cardiomyopathy [36,37] and may be repurposed for PPCM following rigorous evaluation.

Several limitations should be acknowledged. First, the sample size was modest, which may limit statistical power and the generalizability of the findings. Second, ROC analyses were performed without external validation in an independent cohort; therefore, the reported discriminative performance should be considered exploratory. Third, potential confounding factors such as dietary carnitine intake, lactation status, medication use (including heart failure therapy), and metabolic comorbidities were not fully controlled and may have influenced circulating acylcarnitine levels. Finally, the cross-sectional design precludes causal inference and does not allow assessment of temporal metabolic changes during disease progression or recovery. In light of these limitations, longitudinal and multi-center validation studies are warranted to confirm and extend the present findings.

Future research should prioritize longitudinal studies to monitor metabolic changes from pregnancy through postpartum recovery, integrating metabolomics with other omics approaches such as transcriptomics and proteomics. Such a systems-level strategy may identify upstream regulators of carnitine metabolism and reveal novel therapeutic targets. In addition, validation in larger, multi-center cohorts is essential to confirm the diagnostic and prognostic utility of acylcarnitine profiles in PPCM.

## 5. Conclusions

In conclusion, this study demonstrates that postpartum cardiomyopathy is associated with a distinct reprogramming of carnitine metabolism, defined by reduced free and long-chain acylcarnitines and elevated short-chain species. These alterations not only illuminate disease mechanisms but also highlight specific acylcarnitines (C2, C6DC, and C16) as promising biomarker candidates. Targeted metabolomics may represent exploratory biomarker candidates requiring validation in larger cohorts.

## Figures and Tables

**Table 1:** Demographic, echocardiographic and laboratory findings of the PPCM patients and control group.

Variables	Control (n:40)	PPCM Group (n:40)	*p*-Value
Maternal age at diagnosis, years	27.3 ± 7.9	29.2 ± 8.1	0.183
Postpartum presentation, n (%)	13 (32.5)	15 (37.5)	0.092
Primiparity, n (%)	9 (22.5)	10 (25)	0.416
Multifetal pregnancies, n (%)	1 (2.5)	2 (5)	0.080
Gestational hypertension, n (%)	4 (10)	8 (20)	0.058
Diabetes mellitus, n (%)	2 (5)	3 (7.5)	0.196
Thyroid disease, n (%)	3 (7.5)	4 (10)	0.254
Heart rate, bpm	102.2 ± 10.4	115.4 ± 15.2	0.368
LVEDD, mm	46.4 ± 5.3	53.2 ± 5.8	**<0.001**
LVESD, mm	30.5 ± 6.8	39.2 ± 7.1	**<0.001**
IVS, mm	9.2 ± 1.0	9.0 ± 0.9	0.642
PDd, mm	8.9 ± 0.9	9.1 ± 1.0	0.594
LA diameter, mm	34.4 ± 5.1	38.7 ± 6.0	0.231
LVEF, %	60.7 ± 9.2	39.8 ± 7.3	**<0.001**
NYHA function class (III and IV), n (%)	-	14 (35)	
eGFR, mL/min/1.73 m^2^	105.7 ± 22.4	99.8 ± 20.4	0.176
Hemoglobin, g/dL	10.2 ± 2.4	9.8 ± 2.3	0.342
WBC, 10^3^ µL	10.7 ± 5.1	11.6 ± 4.8	0.148
Platelet counts, 10^3^ µL	245.6 ± 70.5	320.0 ± 72.7	0.206
ALT, mmol/L	20.2 ± 3.8	30.4 ± 6.5	0.062
NT-ProBNP, pg/mL	40.4 ± 16.8	608.4 ± 190.6	**<0.001**

Values are presented as mean ± standard deviation (SD). **Abbreviations:** PPCM: postpartum cardiomyopathy; LVEDD: left ventricular end diastolic diameter; LVESD: left ventricular end systolic diameter; IVS: interventricular septum thickness; PDd: posterior wall thickness; LA: left atrium; LVEF: left ventricular ejection fraction; NYHA: New York Heart Association; eGFR: glomerular filtration rate; WBC: white blood cells; ALT: alanine aminotransferase.

**Table 2:** Altered serum carnitine and acylcarnitine profiles in PPCM patients and healthy controls. Values are presented as mean ± standard deviation (SD) and expressed in µmol/L. Between-group comparisons were performed using independent-samples *t*-tests. *p*-values were adjusted for multiple comparisons using the false discovery rate (FDR) method. Statistical significance was defined as FDR-adjusted *p* < 0.05.

Carnitine	Control Group (n:40)	PPCM Group (n:40)	*p*-Value
C0	187.52 ± 57.60	97.40 ± 30.63	**<0.001**
C2	6.498 ± 4.33	23.552 ± 3.82	**<0.001**
C3	0.756 ± 0.40	1.001 ± 005	**<0.001**
C4	0.668 ± 0.34	0.601 ± 0.04	**<0.001**
C4DC	0.054 ± 0.02	0.050 ± 0.00	**<0.001**
C5	0.488 ± 0.18	0.401 ± 0.03	**<0.001**
C5:1	0.050 ± 0.02	0.042 ± 0.01	**<0.001**
C5OH	0.077 ± 0.03	0.083 ± 0.00	**<0.001**
C5DC	0.546 ± 0.30	0.178 ± 0.03	**<0.001**
C6	0.197 ± 0.09	0.143 ± 0.01	**<0.001**
C6DC	0.086 ± 0.04	0.154 ± 0.05	**<0.001**
C8	0.554 ± 0.26	0.362 ± 0.02	**<0.001**
C8:1	0.402 ± 0.22	0.304 ± 0.02	**<0.001**
C8DC	0.029 ± 0.02	0.052 ± 0.02	**<0.001**
C10	0.882 ± 0.45	0.474 ± 0.04	**-**
C10:1	0.577 ± 0.29	0.233 ± 0.01	**<0.01**
C10DC	0.029 ± 0.01	0.034 ± 0.00	**<0.001**
C12	0.202 ± 0.11	0.094 ± 0.01	**<0.01**
C14	0.105 ± 0.06	0.073 ± 0.01	**<0.001**
C14:1	0.140 ± 0.08	0.034 ± 0.00	**<0.001**
C14:2	0.037 ± 0.02	0.021 ± 0.01	**<0.05**
C16	0.312 ± 0.17	0.170 ± 0.01	**-**
C16:1	0.090 ± 0.05	0.022 ± 0.01	**<0.001**
C18	0.124 ± 0.06	0.081 ± 0.01	**<0.001**
C18:1	0.181 ± 0.09	0.024 ± 0.01	**<0.001**
C18:2	0.082 ± 0.04	0.034 ± 0.01	**<0.001**
C18:1 OH	0.034 ± 0.02	0.020 ± 0.00	**<0.05**

Data are expressed in frequencies (percentages) or mean ± standard deviation. **Abbreviations:** C0, free carnitine; C2, acetylcarnitine; C3, propionylcarnitine; C4, butyrylcarnitine; C4DC, succinylcarnitine; C5, isovalerylcarnitine; C5OH, hydroxyisovalerylcarnitine; C5DC, glutarylcarnitine; C6, hexanoylcarnitine; C6DC, adipoylcarnitine; C8, octanoylcarnitine; C8:1, octenoylcarnitine; C10, decanoylcarnitine; C10:1, decenoylcarnitine; C12, dodecanoylcarnitine; C14, tetradecanoylcarnitine; C14:1, tetradecenoylcarnitine; C14:2, tetradecadienoylcarnitine; C16, palmitoylcarnitine; C16:1, palmitoleylcarnitine; C18, stearoylcarnitine; C18:1, oleoylcarnitine; C18:2, linoleoylcarnitine.

**Table 3:** Statistical comparison of significantly altered acylcarnitines in PPCM and controls.

Metabolite (Biochemical Name)	t (df = 78)	FDR-Adjusted *p*-Value	–log10(p)	Cohen’s d	Δ Mean (95% CI), µmol/L
C2 (Acetylcarnitine)	19.748	<0.001	10.342	4.18	17.054 (15.236–18.872)
C3 (Propionylcarnitine)	9.319	<0.001	4.884	0.86	0.245 (0.118–0.372)
C4DC (Succinylcarnitine)	10.936	<0.001	5.732	−1.73	−0.368 (−0.463–0.273)
C14:1 (Tetradecenoylcarnitine)	−6.64	<0.001	3.458	−1.87	−0.106 (−0.131–0.081)
C18:1 (Oleoylcarnitine)	−8.847	<0.001	4.598	−2.45	−0.157 (−0.186–0.128)

Values are reported as the t statistic from an independent-samples *t*-test with degrees of freedom (df = 78). *p*-values were adjusted for multiple comparisons using the false discovery rate (FDR) method and −log10(p) values are shown for reference. Cohen’s d and mean differences (Δ) with 95% confidence intervals (CI) were computed using group means and standard deviations (n = 40 per group). Biochemical nomenclature is standardized throughout.

## Data Availability

The data supporting the findings of this study are available from the corresponding author upon reasonable request.
